# MRI evidence that glibenclamide reduces acute lesion expansion in a rat model of spinal cord injury

**DOI:** 10.1038/sc.2013.99

**Published:** 2013-09-17

**Authors:** JM Simard, PG Popovich, O Tsymbalyuk, J Caridi, RP Gullapalli, MJ Kilbourne, V Gerzanich

**Affiliations:** 1 Department of Neurosurgery, University of Maryland School of Medicine, Baltimore, MD, USA; 2 Department of Pathology, University of Maryland School of Medicine, Baltimore, MD, USA; 3 Department of Physiology, University of Maryland School of Medicine, Baltimore MD, USA; 4 Center for Brain and Spinal Cord Repair and Department of Neuroscience, The Ohio State University College of Medicine, Columbus, OH, USA; 5 Department of Radiology, University of Maryland School of Medicine, Baltimore MD, USA; 6 Department of Surgery, Walter Reed Army Medical Center, Washington, DC, USA

**Keywords:** spinal cord injury, glibenclamide, riluzole, Sur1-Trpm4 channel, MRI, progressive hemorrhagic necrosis

## Abstract

**Study design:**

Experimental, controlled, animal study.

**Objectives:**

To use non-invasive magnetic resonance imaging (MRI) to corroborate invasive studies showing progressive expansion of a hemorrhagic lesion during the early hours after spinal cord trauma and to assess the effect of glibenclamide, which blocks Sur1-Trpm4 channels implicated in post-traumatic capillary fragmentation, on lesion expansion.

**Setting:**

Baltimore.

**Methods:**

Adult female Long–Evans rats underwent unilateral impact trauma to the spinal cord at C7, which produced ipsilateral but not contralateral primary hemorrhage. In series 1 (six control rats and six administered glibenclamide), hemorrhagic lesion expansion was characterized using MRI at 1 and 24 h after trauma. In series 2, hemorrhagic lesion size was characterized on coronal tissue sections at 15 min (eight rats) and at 24 h after trauma (eight control rats and eight administered glibenclamide).

**Results:**

MRI (T2 hypodensity) showed that lesions expanded 2.3±0.33-fold (*P*<0.001) during the first 24 h in control rats, but only 1.2±0.07-fold (*P*>0.05) in glibenclamide-treated rats. Measuring the areas of hemorrhagic contusion on tissue sections at the epicenter showed that lesions expanded 2.2±0.12-fold (*P*<0.001) during the first 24 h in control rats, but only 1.1±0.05-fold (*P*>0.05) in glibenclamide-treated rats. Glibenclamide treatment was associated with significantly better neurological function (unilateral BBB scores) at 24 h in both the ipsilateral (median scores, 9 vs 0; *P*<0.001) and contralateral (median scores, 12 vs 2; *P*<0.001) hindlimbs.

**Conclusion:**

MRI is an accurate non-invasive imaging biomarker of lesion expansion and is a sensitive measure of the ability of glibenclamide to reduce lesion expansion.

## INTRODUCTION

Spinal cord injury (SCI) remains one of the foremost unsolved challenges in medicine. Worldwide, the incidence of SCI ranges from 10 to 83 per million people per year, with half of these patients suffering a complete lesion and one-third becoming tetraplegic.^1^ At present, little can be done to undo or repair the initial damage to spinal cord tissues, but great hope lies in reducing secondary injury processes triggered by the primary injury that increase the damage and worsen clinical outcome.

Histological studies on animal models of SCI have shown that early expansion of a hemorrhagic contusion is a common feature following trauma to the spinal cord. During the hours after a blunt impact, a dynamic process ensues wherein a hemorrhagic contusion slowly enlarges, resulting in the progressive autodestruction of spinal cord tissues.^2–4^ Discrete petechial hemorrhages appear, first around the site of injury and then in more distant areas.^5^ As petechial hemorrhages form and coalesce, the lesion gradually expands, with a characteristic region of hemorrhage that `caps' the advancing front of the lesion. A small hemorrhagic lesion that initially involves primarily the capillary-rich gray matter doubles in size during the first 24 h after injury. The advancing hemorrhage results from delayed progressive catastrophic failure of the structural integrity of capillaries, a phenomenon termed `progressive hemorrhagic necrosis'.^2–4^

Progressive hemorrhagic necrosis has been linked to *de novo* upregulation of sulfonylurea receptor 1 (Sur1)—transient receptor potential melastatin 4 (Trpm4) channels (a.k.a., Sur1-regulated NC_Ca-ATP_ channels)^6^ in microvessels.^4,7,8^ Sur1-Trpm4 channels have been shown to be responsible for the necrotic death of endothelial cells that results in delayed fragmentation of capillaries and formation of petechial hemorrhages. Gene suppression as well as pharmacological blockade of Sur1-Trpm4 channels prevents progressive hemorrhagic necrosis, reduces lesion size and significantly improves neurological function in rodent models of SCI.^7–10^

To date, the magnitude and time course of lesion expansion due to progressive hemorrhagic necrosis have been characterized in rat models by measuring the amount of extravasated blood in the spinal cords of animals that were killed at different times after trauma.^4^ Although an important measure of injury, quantifying the amount of extravasated blood may not accurately reflect the actual volume of compromised tissue, as this technique cannot distinguish between an increase in the number of erythrocytes within a given volume of tissue versus an actual increase in volume of contused tissue affected by hemorrhage and edema. We hypothesized that magnetic resonance imaging (MRI) could be used to assess early lesion expansion non-invasively and to independently corroborate the existence of early lesion expansion due to progressive hemorrhagic necrosis after spinal cord trauma. Here, we used serial MRI scans obtained at 1 and 24 h after trauma to characterize lesion expansion, and we validated our MRI measurements by comparing them with measurements based on coronal tissue sections in a different group of rats with the same injury. In these experiments, we also examined the effect of glibenclamide on lesion expansion and on neurological function.

## MATERIALS AND METHODS

### Ethics statement

We certify that all applicable institutional and governmental regulations concerning the ethical use of animals were followed during the course of this research. Animal experiments were performed under a protocol approved by the Institutional Animal Care and Use Committee (IACUC) of the University of Maryland, Baltimore, and in accordance with the relevant guidelines and regulations as stipulated in the United States National Institutes of Health Guide for the Care and Use of Laboratory Animals. All efforts were made to minimize the number of animals used and their suffering. In accordance with `good laboratory practice', different investigators blinded to injury-group conducted behavioral tests and analyzed the data.

### Subjects and experimental series

These experiments were conducted on new series of animals distinct from those previously reported from this laboratory. Thirty-six female Long–Evans rats (250–300 gm; Harlan, Indianapolis, IN, USA) were used in two experimental series (Table 1). In series 1, 12 rats underwent an SCI (see below), six were administered glibenclamide (see below) and six served as untreated controls; these 12 rats were studied using MRI at 1 and 24 h after trauma, after which they were euthanized. In series 2, 24 rats underwent an SCI; eight were euthanized at 15 min; of the remaining 16 rats, eight served as untreated controls and eight were administered glibenclamide; these 16 rats were assessed for locomotor function at 24 h, after which they were euthanized to obtain tissue sections for measurements of the hemorrhagic lesion area at the epicenter.

### Sample size calculation

Previous experiments with the model used here suggested that an effect size (Cohen's d) of 2.27 (means, 2 vs 1 μl; pooled s.d., 0.44 μl) might be expected for hemorrhage volumes with vehicle vs glibenclamide treatments.^4^ Sample size calculation for a 2-sample comparison (α=0.05; 2-tailed), an effect size of 2.27, and a desired power of 90% indicated a minimum sample size of six per group.

### Rat model of SCI

A unilateral impact to the cervical spinal cord at C7 was calibrated to produce ipsilateral but not contralateral primary hemorrhage, resulting in severe SCI, as described in detail previously.^4,8–10^ Female Long–Evans rats (~11 weeks; 250–300 gm; Harlan) were anesthetized (Ketamine, 60 mg kg^−1^ plus Xylazine, 7.5 mg kg^−1^, intraperitoneally (IP)). On the left side, the entire lamina of C7 and the dorsal half of the pedicle of C7 were removed. The dura was exposed by removing the interlaminar ligaments rostral and caudal to the lamina of C7 and any remaining ligamentum flavum. The guide tube containing the impactor (1.55-mm tip diameter, 57 mm length, 1.01 gm)^11^ was angled 5° medially and was positioned using the manipulator arm of the stereotaxic apparatus. The impactor was activated when struck by a 10-gm weight dropped from a height of 25 mm inside the guide tube (velocity, 0.7 m s^−1^). After injury, rats were nursed on a heating pad to maintain a rectal temperature of ~37 °C and were given 10 ml of glucose-free normal saline subcutaneously. Food and water were placed within easy reach to ensure that the rats were able to eat and drink without assistance. Bladder function was assessed 2–3 times daily by observing for urination, and, if needed, manual emptying of the bladder was carried out using the Credé maneuver.

### Treatment

Treatment consisted of the following: (1) administering a single loading dose of glibenclamide (10 μg kg^−1^) or an equivalent volume of vehicle IP, at 5 min after trauma; and (2) implanting a mini-osmotic pump (Alzet 2001, 1.0 μl h^−1^; Alzet Corp., Cupertino, CA, USA) for continuous subcutaneous infusion of glibenclamide (200 μg ml^−1^) beginning at the end of surgery, resulting in delivery of 200 ng h^−1^ or an equivalent volume of vehicle subcutaneously until euthanasia. This dosing regimen was found previously to significantly ameliorate progressive hemorrhagic necrosis^8–10^ without producing clinically relevant hypoglycemia or any other toxicity.^4,12,13^ Unlike our previous study, in which a 3-h treatment delay was used,^9^ here we used an early treatment time because our principal goal was to validate MRI for measuring early lesion expansion. The formulation of glibenclamide (#G2539; Sigma, St Louis, MO, USA) in dimethyl sulfoxide (DMSO) and the preparation of mini-osmotic pumps have been described in detail.^14^ Briefly, a stock solution was prepared by placing 25 mg of glibenclamide into 10 ml of DMSO. The solution used for the loading dose was prepared by adding 4 μl of stock solution to 1 ml unbuffered normal saline. The solution for infusion was prepared by taking 2.3-ml unbuffered normal saline, adding 4 μl of 10 n NaOH (undiluted Fixanal; Riedel-deHaën, Seelze, Germany), and then adding 200-μl stock solution, in that order in order to prevent precipitation of the drug. Control animals received vehicle solution prepared as above but without glibenclamide.

### Measurements of primary and secondary hemorrhage

In series 2, at the designated time after injury (15 min for primary hemorrhage; 24 h for primary plus secondary hemorrhage), the rat was anesthetized with sodium pentobarbital (100 mg kg^−1^), a thoracotomy was performed and the rat was transcardially perfused with normal saline (50 ml) at a pressure of 100 cm H_2_O. The spinal cord was collected and sectioned coronally through the epicenter of injury, and the face of the epicenter was imaged at high resolution (1200 dpi) using a flat-bed scanner (Epson Perfection 2450 Photo).

### Locomotor function

Basso, Beattie and Bresnahan (BBB) scores were determined as described,^15^ except that modifications were introduced to allow more accurate assessment of the functional asymmetry associated with hemicord injury. We refer to this modified functional assessment as modified (unilateral) BBB scores.^9,10^

### Magnetic resonance imaging

In series 1, all rats were imaged 1 and 24 h after trauma; four control rats also were imaged 6 h after trauma. The delay of 1 h in obtaining the `baseline' MRI was necessitated by logistical considerations, principally the distance between the site where injuries were produced and the site where MRIs were obtained. MRI was performed on a 3.0 Tesla Siemens Trio MR scanner equipped with 18 receiver channels and high performance gradients (200 mTm^−1^ms^−1^). An eight-channel wrist coil was used to image the rats. Anesthetized (Ketamine, 60 mg kg^−1^ plus Xylazine, 7.5 mg kg^−1^, IP) animals were placed in the supine position with the `sweet spot' of the coil centered on the cervical spine of the rat. The depth of anesthesia was monitored continuously during the imaging session.

The imaging sequences consisted of localizers in the three orthogonal planes. Following localization, T1-weighted magnetization prepared rapid acquisition gradient echo (MP-RAGE) images were acquired in the axial plane with adequate coverage of the injury using 64 slices over a field of view of 53 mm × 62 mm and an interpolated pixel resolution of 448 × 512. The imaging parameters were as follows: inversion time TI=900 ms and TE/TR 4 ms/1900 ms for an acquisition time of 3 min and 53 s using an acceleration factor of 2. T2-weighted 3D spin-echo images were then acquired in the sagittal and the axial plane. For both planes, the imaging parameters were as follows: TE/TR=93 ms/1000 ms, 64 slices, slice thickness 0.3 mm over a field of view of 45 mm × 90 mm at an interpolated pixel resolution of 312 × 640 for a total acquisition time of 5 min and 24 s using parallel imaging with an acceleration factor of 2.

All images were reconstructed in three planes to visualize the extent of the lesion. The hemorrhagic portion of the contusion was identified as a hypointense region (due to the presence of hemoglobin) within the cord on T2-weighted images. These regions were demarcated on each slice, and the total volume of the lesion was determined.

### Data analysis

T2-hypodensity volumes at 1 versus 24 h were analyzed using Student's paired *t*-test. Areas of hemorrhagic contusion on tissue sections at the epicenter in three groups of rats (15 min untreated, 24-h vehicle, 24-h glibenclamide) were analyzed using AVOVA, with Bonferroni *post hoc* comparisons. Modified (unilateral) BBB scores in two groups of rats (vehicle vs glibenclamide) were analyzed using the Mann–Whitney *U*-test.

## RESULTS

**Figure 1:**
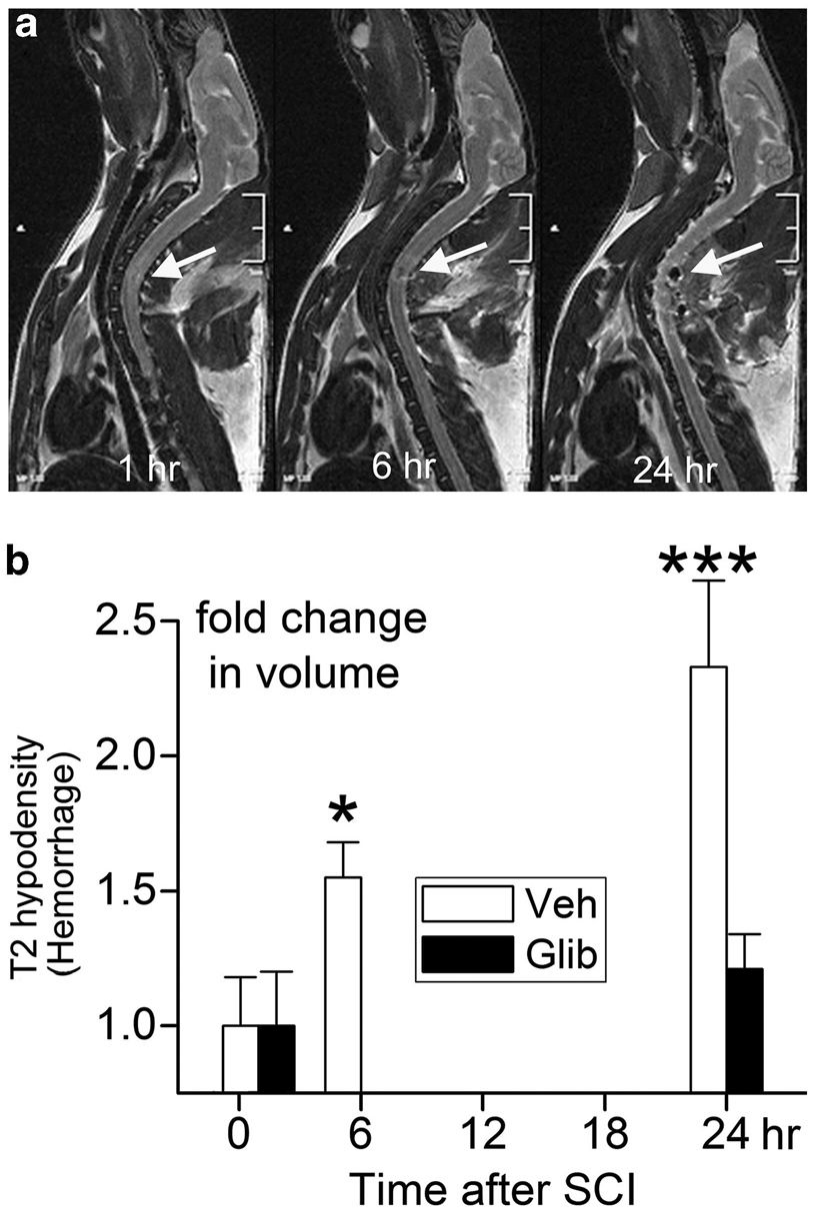
Progressive expansion of hemorrhagic contusion assessed using MRI. (**a**) Serial MRIs of a control rat obtained at 1, 6 and 24 h after trauma, showing progressive enlargement of the T2 hyperdensity due to the presence of increasing amount of hemoglobin; the data shown are representative of six rats. (**b**) Fold-change in volumes of T2 hyperdensity measured at the times indicated in control rats (Veh; empty bars) versus glibenclamide-treated rats (Glib; filled bars); mean±s.e.; six rats per group; *P<0.05; ***P<0.001.

In series 1, we evaluated the expansion of the hemorrhagic contusion using serial MRI scans obtained at 1 and 24 h after trauma, with the T2 hypodensity being taken as a measure of extravasated blood.^16–19^ In control rats, a small T2 hypodensity was evident at the first examination, obtained 1 h after trauma (Figure 1a). Subsequent imaging obtained at 6 h (*n*=4) and 24 h (*n*=6) confirmed progressive enlargement of the hemorrhagic lesion (Figure 1a). In six control rats, the volume (mean±s.e.) of the T2 hypodensity at 1 h after trauma was 1.52±0.37 μl, and, after 24 h, the volume increased 2.3-fold to 3.55±0.87 μl (*P*<0.01) (Figure 1b). By contrast, in six rats treated with glibenclamide 5 min after trauma, the volume of the T2 hypodensity at 1 h was 0.51±_0.21_ μl, and, after 24 h, the volume increased 1.2-fold to 0.59±0.24 μl (*P*=0.43) (Figure 1b).

In series 2, we evaluated the size of the hemorrhagic contusion using images of tissue sections taken through the epicenter of injury at 15 min and at 24 h. We considered the hemorrhagic area observed at 15 min to represent the primary hemorrhage due to the trauma and the hemorrhagic area observed at 24 h to represent the primary hemorrhage plus the secondary hemorrhage, the latter attributable to progressive hemorrhagic necrosis.

**Figure 2:**
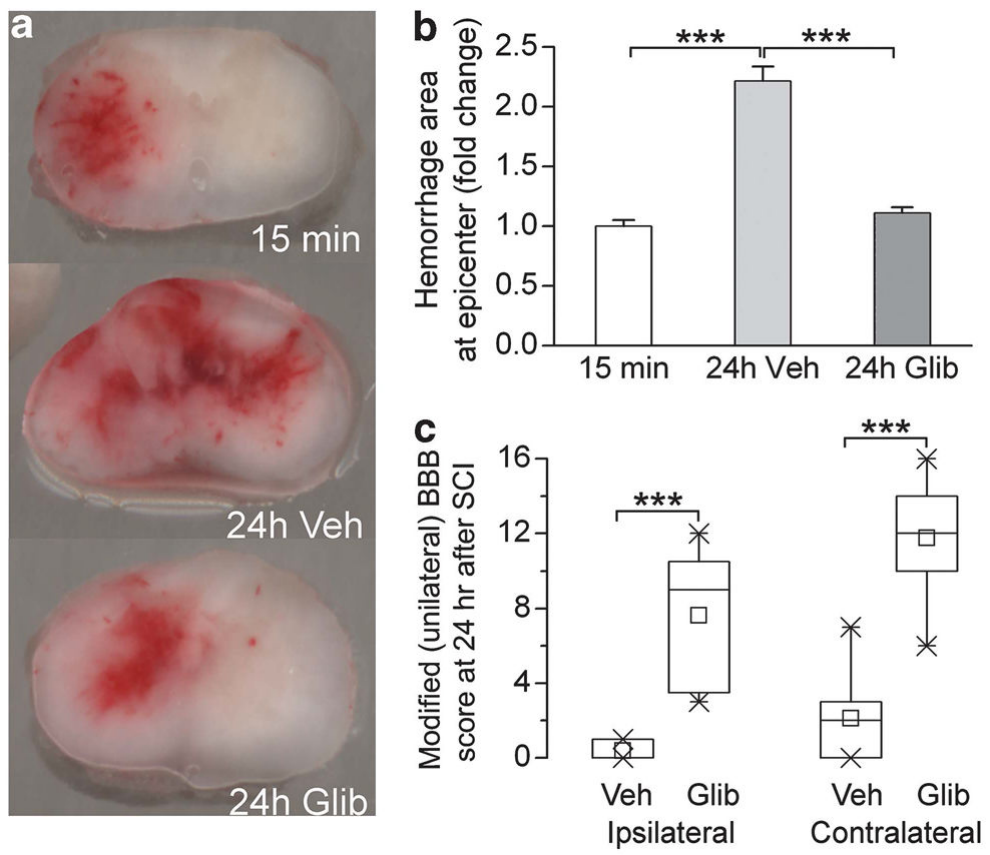
Progressive expansion of hemorrhagic contusion assessed from tissue sections at the epicenter. (**a**) Representative coronal tissue sections of perfusion-cleared but otherwise unprocessed spinal cords through the epicenter of injury 15 min (top) and 24 h (middle and bottom) after impact, in control rats (top and middle), and in a glibenclamide-treated rat (bottom); the data shown are representative of eight rats per group. (**b**): Fold-change in areas of hemorrhage at the epicenter in the three groups of rats depicted in (*a*); mean±s.e.; eight rats per group; ***P<0.001. (**c**) Box plots showing modified (unilateral) BBB scores for the ipsilateral and contralateral hindlimbs at 24 h, for the two groups of rats depicted in (b, 24 h Veh and 24 h Glib); box, 25th and 75th percentiles; ×, 1st and 99th percentiles; line, median; small square, mean.

At 15 min, a contusion was apparent that was confined largely to the ipsilateral hemicord, mostly the gray matter (Figure 2a, upper). In control rats, the hemorrhagic contusion at 24 h invariably involved a larger area, typically extending to the contralateral side (Figure 2a, middle). By contrast, in rats administered glibenclamide 5 min after trauma, the hemorrhagic contusion at 24 h typically occupied an area comparable to that observed at 15 min (Figure 2a, lower). Quantification of the hemorrhagic area showed that, in control rats, lesions were 2.2±0.12-fold larger at 24 h compared with that at 15 min By contrast, in glibenclamide-treated rats, lesions were 1.1±0.05-fold larger at 24 h compared with that at 15 min (Figure 2b).

Commensurate with the favorable effect of glibenclamide on hemorrhagic lesion area, assessment of modified (unilateral) BBB scores at 24 h showed that glibenclamide was associated with significantly better neurological function. Median scores were 0 vs 9 (P<0.001) for the ipsilateral hindlimb and 2 vs 12 (P<0.001) for the contralateral hindlimb, for the control vs glibenclamide groups, respectively (Figure 2c).

## DISCUSSION

The principal finding of the present study is that, based on measurements of MRI T2-lesion volume and measurements of hemorrhagic lesion area, there is a 2- to 2.5-fold increase in the hemorrhagic contusion that takes place during the first 24 h after blunt impact trauma to the spinal cord, in good agreement with previous measurements based on tissue hemoglobin.^4^ Previous histological and MRI studies in rats have characterized spinal cord lesions at various times after injury, but relatively few have examined temporal and spatial characteristics of lesion progression during the first hours after injury. To our knowledge, the earliest study addressing this question (weight drop; midline, lower thoracic/upper lumbar) reported that, on H&E-stained sections, intramedullary hemorrhages involved an aggregate of 11% of the spinal cord area at the level of maximal bleeding immediately after trauma and that this increased 2.5-fold to 28% after 8 h.^20^ In our previous study (weight drop; lateral C7), we reported a twofold increase in the amount of extravasated blood in tissues from the epicenter during the first 12 h after trauma.^4^ In an MRI study (0.5 mm compression for 30 msec; T7), the T2-lesion volume was found to expand B1.5-fold over 5.5 h.^16^ Together with the present study, these various animal studies using different methods establish conclusively the existence of significant lesion expansion during the early hours after blunt impact to the spinal cord. Recently, lesion expansion also was demonstrated using serial MRI scans in humans with cervical SCI.^21^ The importance of these observations lies in the hope that, if early lesion expansion can be halted, patients with acute SCI may suffer the least possible secondary injury.

The model of cervical hemicord impact that we used here is particularly well suited to examining the expansion of the primary injury to the contralateral side. In this model, care is taken to obtain a primary hemorrhage exclusively ipsilateral to the site of impact. This model emphasizes the distinction between primary and secondary hemorrhage, and it shows the influence of secondary hemorrhage on outcome.^8^ When primary hemorrhage is located exclusively unilaterally, subsequent spread of the hemorrhagic lesion to the contralateral side during the ensuing hours—by definition, secondary hemorrhage—is readily discerned both histologically and functionally. Moreover, if the primary hemorrhage is confined to one side and if secondary expansion of the hemorrhagic lesion to the contralateral side is prevented, the magnitude and importance of secondary hemorrhage is readily appreciated. By contrast, with bilateral primary hemorrhage, neurological dysfunction due to secondary hemorrhage is more difficult to detect. When the primary hemorrhage already occupies most of a spinal segment, expansion laterally of secondary hemorrhage may be limited or functionally mute. Rostro-caudal expansion would still occur but, depending on the spinal cord level involved, for example, cervical vs thoracic, this may be difficult to detect neurologically.

An important aspect of the present study is that it confirmed previous observations that early lesion expansion can be prevented by blocking either of the two subunits of the Sur1-Trpm4 channel.^6^ Lesion expansion is prevented by blocking Sur1, either pharmacologically with glibenclamide or repaglinide,^4^ or by gene suppression with antisense oligodeoxynucleotide or gene deletion of *Abcc8*,^8^ or by blocking Trpm4, either pharmacologically with flufenamic acid^7^ or riluzole,^9^ or by gene suppression with antisense oligodeoxynucleotide or gene deletion of *Trpm4*.^7^

In the present study examining acute outcomes at 24 h, in three other series from our laboratory examining outcomes at 1 or 6 weeks,^4,8,10^ and in one series from an independent laboratory,^22^ glibenclamide treatment beginning shortly after trauma was found to be highly effective in reducing lesion size and improving neurological function. In a 6th series of rats with outcomes evaluated at 6 weeks, treatment at the clinically more relevant time of 3 h after trauma also was found to be highly beneficial.^9^ As might be expected, the magnitude of the benefit observed with glibenclamide depends on the magnitude of the primary injury,^10^ but all studies to date examining functional outcome and lesion size at 6 weeks have demonstrated a significant treatment effect, regardless of the initial severity.^8–10^

In the 6 series to date with glibenclamide, drug was delivered by constant subcutaneous infusion. From a pharmacokinetic perspective, cutaneous delivery of glibenclamide is highly effective for maintaining steady plasma levels, is superior to enteral administration and appears to be equivalent to intravenous (IV) administration.^23^ Constant subcutaneous infusion of glibenclamide was used in the preclinical studies as a convenient alternative to constant IV infusion, as is used with injectable glibenclamide (RP-1127) in clinical trials for other CNS indications (ClinicalTrials.gov identifiers: NCT01454154; NCT01268683; NCT01794182). In the animal studies, no clinically relevant hypoglycemia or other toxicity has been detected with infusions of 200 ng h^−1 4,12,13^ or 400 ng h^−1^.^24^ In a Phase I trial of RP-1127 in 16 healthy volunteers (ClinicalTrials.gov identifier: NCT01132703), a 3-day IV infusion (125 mg/h) produced no clinically significant hypoglycemia or other serious adverse event (S. Jacobson, personal communication).

A recent comprehensive review^25^ shows how difficult it can be to translate preclinical trials on acute pharmacotherapeutic neuroprotection to clinical trials in SCI. Among numerous challenges, consideration must be given to the accumulated preclinical data on any proposed therapeutic agent as well as the effect of that agent on surrogate end points and functional outcome. The work reported here shows that early MRI may be a useful imaging biomarker of lesion expansion, a phenomenon that is prominent in human SCI,^21^ and that may serve as a useful proxy for treatment efficacy.

Riluzole has been found to be efficacious in preclinical models of SCI,^9,26,27^ may have a beneficial effect on motor outcome in cervical SCI, as recently reported in a small open-label Phase I clinical trial,^28^ and is planned for study in a Phase II clinical trial of acute SCI (Clinical Trials.gov identifier, NCT01597518). A recent preclinical study using a rat model of SCI, which was so severe as to have attendant mortality, compared treatment with riluzole (2.5 mg kg^−1^ IP every 12h × 1 week) vs glibenclamide (200 ng h^−1^ continuous subcutaneous infusion −1 week), starting 3 h after trauma.^9^ This study found that glibenclamide is superior to riluzole in terms of both toxicity and efficacy. Riluzole exhibits a peculiar, dose-limiting CNS toxicity in the context of CNS trauma that is absent in non-injured controls: in SCI, mortality rates of 0, 8 and 70% were observed with 4, 6 and 8 mg kg^−1^ IP every 12 h, respectively.^27^ Riluzole is a pleiotropic drug with inhibitory effects on sodium channels, glutamatergic pathways and Trpm4.^9^ However, the near-identity of the phenotypes observed with riluzole^9^ compared with gene suppression of Sur1^8^ as well as gene suppression of Trpm4^7^ suggests that a major effect of riluzole in SCI is via inhibition of the Sur1-Trpm4 channel. Glibenclamide appears to be a safer choice than riluzole for targeting the Sur1-Trpm4 channel in acute CNS injury.

The present study has important shortcomings. First, it was not designed as an efficacy study, but rather to demonstrate the concept that MRI could be used to measure progressive lesion expansion in a rat model of SCI. Thus, the number of subjects studied was small, although the specific number was determined by a power analysis. The most important shortcoming was that the first MRI was obtained 1 h after trauma, whereas treatment with glibenclamide was administered 5 min after trauma. The long delay in obtaining the MRI was necessitated by logistical considerations, principally the distance between the site where injuries were produced and the site where MRIs were obtained. We believe that this problem with study design is the reason for the apparently smaller `baseline' lesions in glibenclamide versus control groups, even though both groups were injured in the same way. Further experiments will be needed to examine MRIs at earlier times. However, if our interpretation is correct, it suggests that `hyper-early' treatment with glibenclamide may be highly desirable for maximally improving outcome.

In conclusion, the present study reaffirms that early enlargement of the hemorrhagic contusion is an important factor determining outcome in SCI, and that reducing early enlargement of the hemorrhagic contusion with glibenclamide can contribute significantly to improved functional outcome.

## Figures and Tables

**Table 1:** Experimental series

	Treatment	Intervention		
		15 min	1 h	24 h
*Series 1*				
Group 1 (*n* = 6)	Control		MRI	MRI euthanize
Group 2 (*n* = 6)	Glibenclamide		MRI	MRI euthanize
*Series 2*				
Group 1 (*n* = 8)	Control	Euthanize section spinal cord		
Group 2 (*n* = 8)	Control			mBBB score euthanize section spinal cord
Group 3 (*n* = 8)	Glibenclamide			mBBB score euthanize section spinal cord

Abbreviations: mBBB, modified (unilateral) Basso, Beattie and Bresnahan; n, number of rats
